# Impact of ambient temperature on energy cost and economical speed during level walking in healthy young males

**DOI:** 10.1242/bio.035121

**Published:** 2018-07-03

**Authors:** Masahiro Horiuchi, Yoko Handa, Yoshiyuki Fukuoka

**Affiliations:** 1 Division of Human Environmental Science, Mt. Fuji Research Institute, Kamiyoshida, 5597-1 Fuji yoshida City, Yamanashi 4030005, Japan; 2 Faculty of Health and Sports Science, Doshisha University, Tatara 1-3, Kyotanabe, Kyoto 6100394, Japan

**Keywords:** Economical speed, Heart rate, Hot environment, Energy expenditure, Pulmonary ventilation

## Abstract

We measured oxygen consumption and carbon dioxide output during walking [per unit distance (*Cw*) values] for 14 healthy young human males at seven speeds from 0.67 to 1.67 m s^−1^ (4 min per stage) in thermoneutral (23°C), cool (13°C), and hot (33°C) environments. The *Cw* at faster gait speeds in the 33°C trial was slightly higher compared to those in the 23°C and 13°C trials. We found the speed at which the young males walked had a significant effect on the *Cw* values (*P*<0.05), but the different environmental temperatures showed no significant effect (*P*>0.05). Economical speed (ES) which can minimize the *Cw* in each individual was calculated from a U-shaped relationship. We found a significantly slower ES at 33°C [1.265 (0.060) m s^−1^ mean (s.d.)] compared to 23°C [1.349 (0.077) m s^−1^] and 13°C [1.356 (0.078) m s^−1^, *P*<0.05, respectively] with no differences between 23°C and 13°C (*P*>0.05). Heart rate and mean skin temperature responses in the 33°C condition increased throughout the walking trial compared to 23°C and 13°C (all *P*<0.05). These results suggest that an acutely hot environment slowed the ES by ∼7%, but an acutely cool environment did not affect the *Cw* and ES.

## Abstract (precis)

**Summary:** Energy cost of walking in a hot environment was greater than in a comfortable environment. Thus, to prevent heat related injury, walking speed should be reduced in a hot environment.

## INTRODUCTION

It is known that there is a U-shaped relationship between energy expenditure (EE) per unit of distance, calculated by oxygen consumption 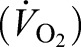 and carbon dioxide output 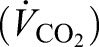 during walking, and gait speed (Horiuchi et al., 2016, 2017). This indicates that there is also a particular gait speed in each individual which can minimize the energy cost of walking; the so-called economical speed (ES) (Horiuchi et al., 2016; Saibene, 1990; Wezenberg et al., 2013). Several factors, including, but not limited to, aging (Malatesta et al., 2003; Mian et al., 2006), load carriage (Abe et al., 2008), changes in body mass (Peyrot et al., 2012) and uphill gradients (Abe et al., 2008) have all been shown to potentially contribute to the impaired energy cost of walking (*Cw*). Additionally, a recent study focused on the effect of environmental factors, e.g. acute hypoxic exposure, on *Cw* and ES and found that severe hypoxia of ∼11% O_2_ impaired the energy cost and slowed ES during level walking (Horiuchi et al., 2016). However, it is unclear whether other environmental factors, such as hot or cool environments, which are frequently encountered during our daily activities and during sports may affect the energy cost of walking and/or ES, seems not to be fully resolved.

Several studies have found that 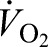 during submaximal exercise does not change in hot conditions compared to a thermoneutral or cool environment (Hayashi et al., 2006; Nybo and Nielsen, 2001a; Périard and Racinais, 2016), suggesting no effect on EE during walking in a hot environment. However, Nybo and Nielsen have also reported that 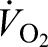 and 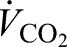 progressively increased during submaximal exercise in a hot environment, while they (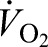 and 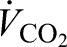) were unchanged throughout the submaximal exercise in thermoneutral trial (Nybo and Nielsen, 2001a). This may also indicate that EE would potentially increase in the latter period during walking.

Given this background, we sought to investigate whether the cost of human locomotion, including an individual's ES, may be affected by different ambient temperature (T_a_) values. We hypothesized that *Cw* at faster gait speeds during walking would be higher in a hot environment than in thermoneutral conditions. These higher *Cw* levels may affect the quadratic function curve (U-shaped curve) and result in a slower ES in a hot environment. To test this hypothesis, we set three T_a_ conditions: thermoneutral (23°C), cool (13°C) and hot (33°C) conditions, and investigated the energy cost of walking.

## RESULTS

Table 1 shows the physiological variables at resting baseline under the three conditions. Cardiorespiratory variables showed equivalent values among the conditions (all *P*>0.05), while heart rate (HR) was significantly higher in the 33°C trial compared to the 23°C and 13°C trials. There were significant differences in mean skin temperature (T_sk_) among the three different environmental conditions. All experimental conditions reduced body weight slightly (−221±47 g at 23°C, −93±39 g at 13°C, and −507±58 g at 33°C, respectively) with significant differences between the conditions (all *P*<0.05).

**Table 1.**
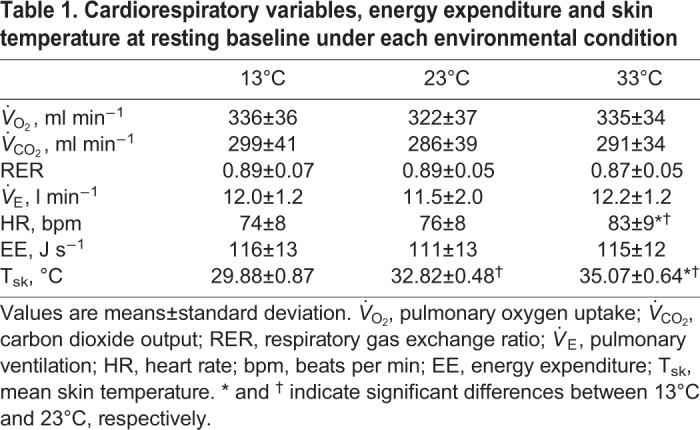
**Cardiorespiratory variables, energy expenditure and skin temperature at resting baseline under each environmental condition**

**Fig. 1.**
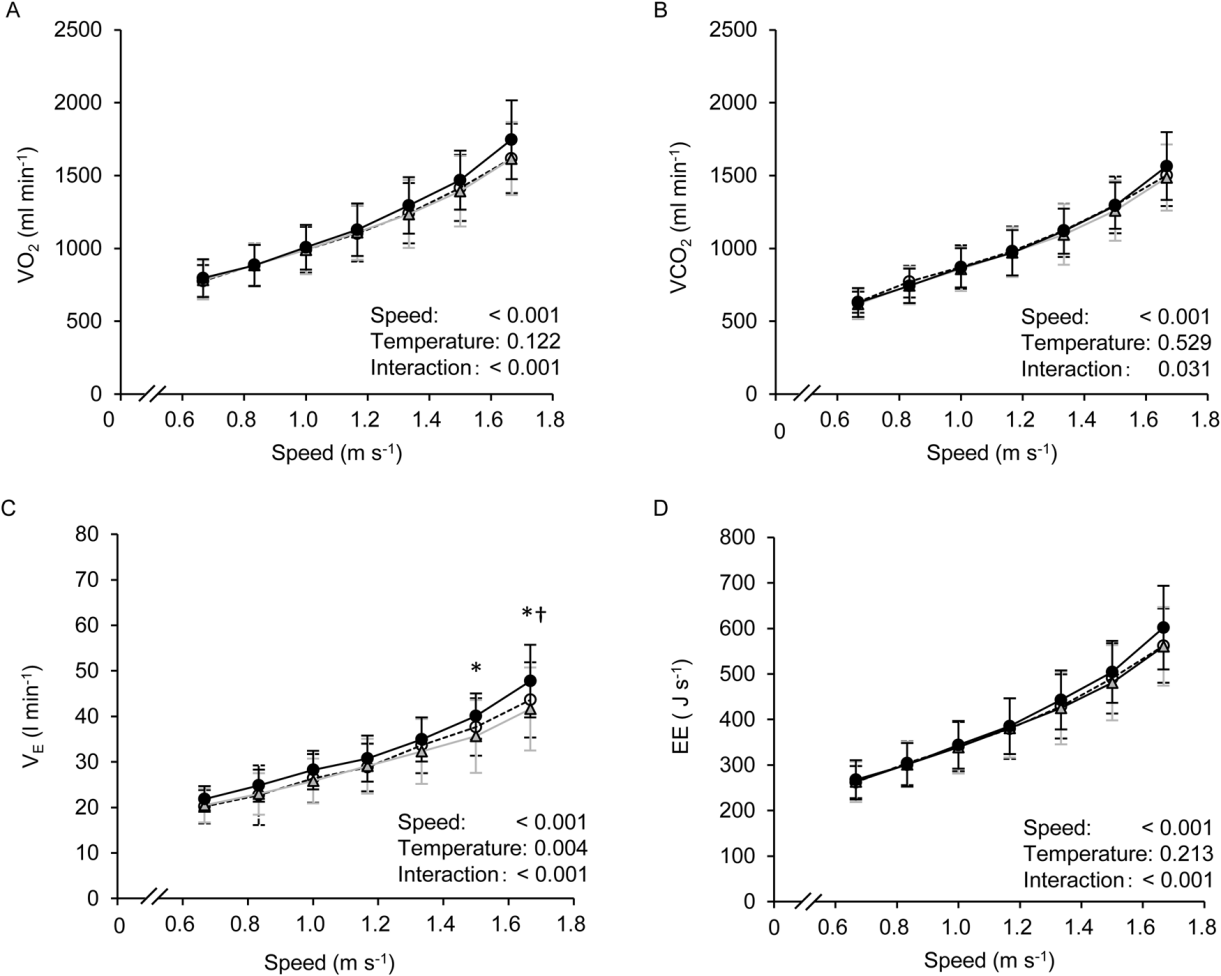
**Changes in 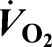 (A), 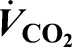 (B), 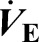 (C), and EE (D) at all gait speeds in thermoneutral (○; 23°C), cool (Δ; 13°C), and hot (●; 33°C) conditions.** Values are means±standard deviation (s.d.). 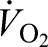 oxygen uptake; 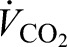, carbon dioxide output; 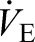, pulmonary ventilation; EE, energy expenditure. *P<0.05 between 33°C and 13°C, ^†^*P*<0.05 between 33°C and 23°C.

The time course changes in 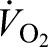, 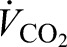, pulmonary ventilation 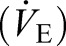 and EE are shown in Fig. 1. All parameters linearly increased in accordance with increasing gait speed. A significant main effect of gait speed and interaction (speed×temperature) was observed in all of these variables; moreover, a significant effect of T_a_ on 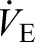 was observed. A Bonferroni post-hoc test revealed that 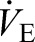 at 1.5 m s^−1^ at 33°C was significantly higher than that at 13°C; moreover, 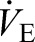 at 1.5 and 1.67 ms^−1^ at 33°C was significantly higher compared to both the 23°C and 13°C trials. In contrast, there was no main effect of T_a_ in 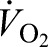, 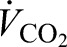 or EE; however, it appeared that these three parameters at the fastest gait speed at 33°C showed slightly higher values compared to the other two conditions though with no statistical differences.

**Fig. 2.**
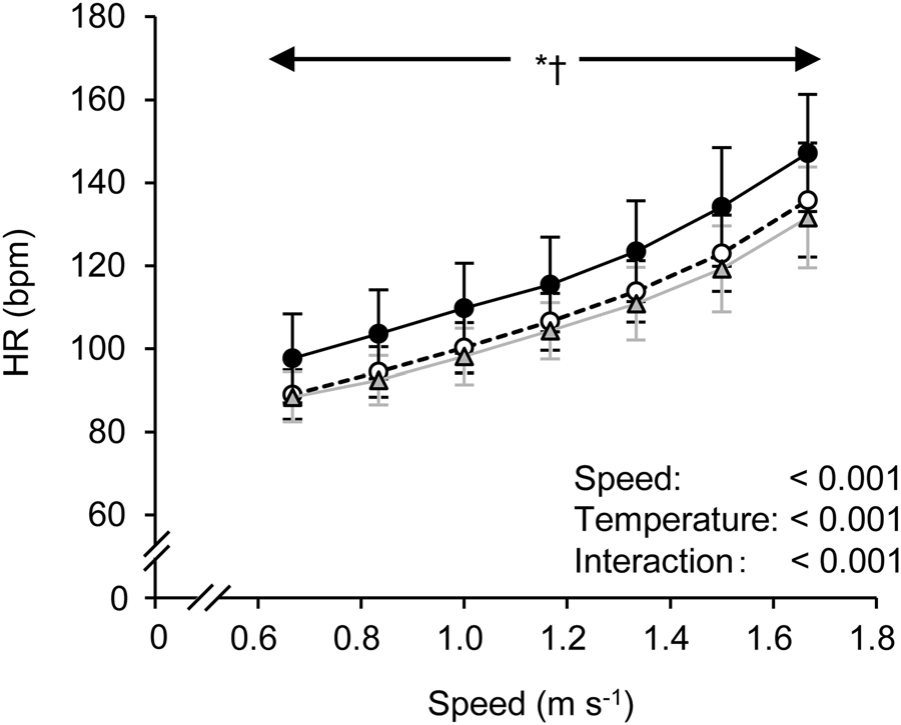
**Changes in HR at all gait speeds in thermoneutral (○; 23°C), cool (Δ; 13°C), and hot (●; 33°C) conditions.** Values are means±s.d. HR, heart rate. * and ^†^ indicate the same as in Fig. 1.

Fig. 2 shows the time course changes in HR across conditions. HR linearly increased in accordance with increasing gait speed. A significant main effect of gait speed, of T_a_ and an interaction (speed×temperature) was observed. A Bonferroni post-hoc test revealed that HR at 33°C was significantly higher throughout walking compared to the other two conditions.

**Fig. 3.**
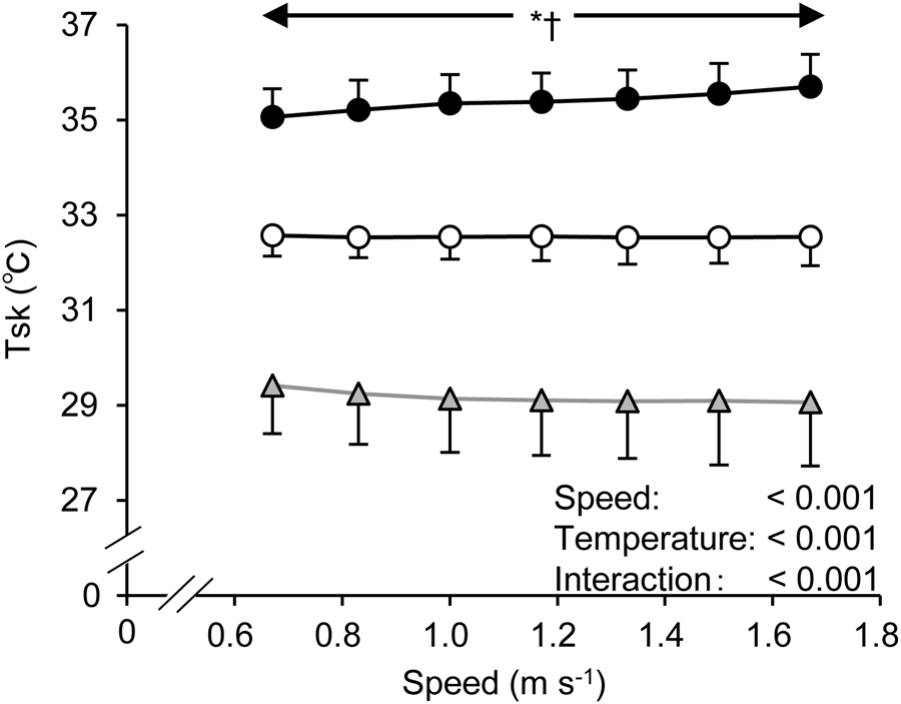
**Changes in T_sk_ at all gait speeds in thermoneutral (○; 23°C), cool (Δ; 13°C), and hot (●; 33°C) conditions.** Values are means±s.d. T_sk_, mean skin temperature. * and ^†^ indicate the same as in Fig. 1.

Fig. 3 shows the time course changes in T_sk_ during walking under three different conditions. T_sk_ at 23°C remained unchanged throughout the walking, while it slightly decreased at 13°C and it slightly increased at the end of walking at 33°C. Throughout the walking, there were significant differences in T_sk_ at all speeds.

**Fig. 4.**
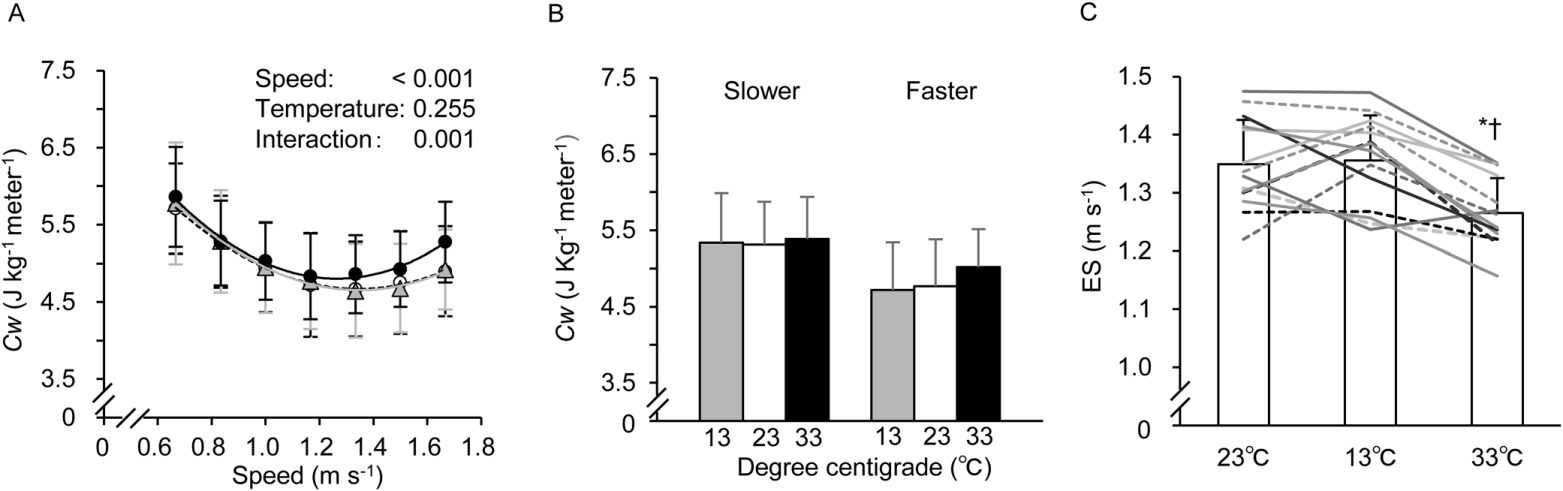
**Changes in *Cw* (A), averaged *Cw* during slower and faster three stages (B), and comparisons of the economical speed (C) in three environmental conditions.** Values of plots (A) and bar graphs (B,C) are means±s.d. In A, each mark indicates the same as in Fig. 1, and each line curve was obtained using a quadratic function. In C, each line graph indicates an individual data of the ES. *Cw*, energy cost of walking; ES, economical speed. * and ^†^ indicate the same as in Fig. 1.

Changes in *Cw* among three environmental conditions are shown in Fig. 4A. A significant main effect of gait speed and interaction (speed×temperature) was observed, while there was no main effect of T_a_. Fig. 4B shows the averaged *Cw* during the slower three gait speeds (between 0.67 and 1.00 m s^−1^) and faster three gait speeds (between 1.33 and 1.67 m s^−1^). One-way repeated ANOVA failed to find a significant difference among conditions (*P*=0.067). The difference in averaged *Cw* at the faster gait speeds between 33°C (5.02±0.49 J kg^−1^ m^−1^) and 23°C (4.77±0.61 J kg^−1^ m^−1^) was ∼5.0%, and the difference between 33°C and 13°C (4.72±0.62 J kg^−1^ m^−1^) was ∼6%. Meanwhile, almost equivalent values were found for *Cw* during the slower gait speeds in all conditions (*P*>0.05). In the 33°C trial, ES was significantly slowed (1.265±0.060 m s^−1^) compared to the 23°C (1.349±0.077 m s^−1^) and 13°C (1.356±0.078 m s^−1^) trials (both *P*<0.05), while the ES was equivalent between these two conditions (*P*>0.05) (Fig. 4C). In regard to the values of coefficients *a* and *b*, calculated using the quadratic function curve, there were no significant differences in coefficient *a* (2.282±0.889 at 23°C, 2.417±1.107 at 13°C, and 2.867±0.746 at 33°C) or coefficient *b* (−6.137±2.360 at 23°C, −6.518±3.024 at 13°C, and −7.245±1.954 at 33°C) on average (all *P*>0.05).

**Fig. 5.**
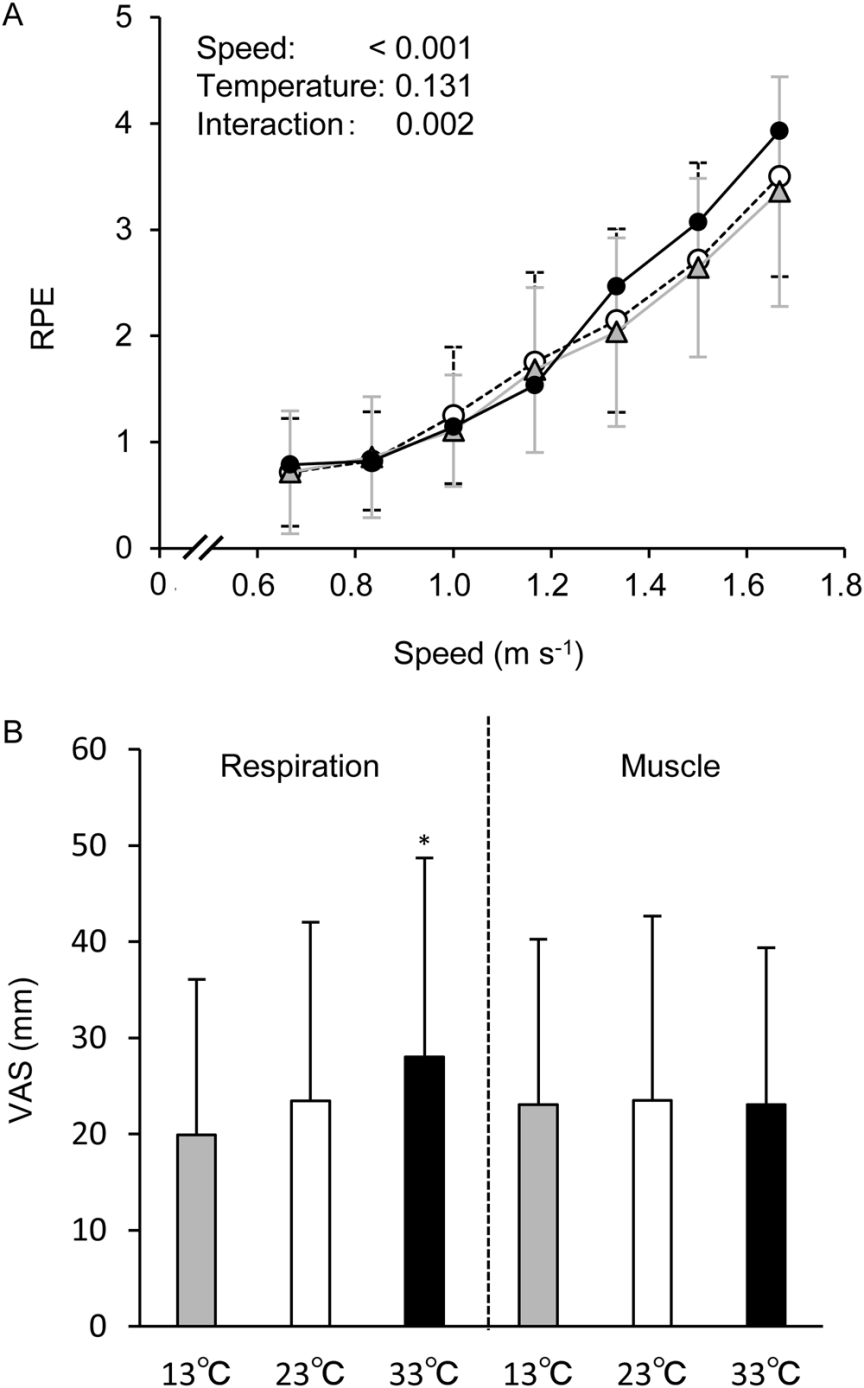
**Changes in RPE (A) and VAS (B) at all gait speeds in three environmental conditions.** Values are means±s.d. RPE, rate of perceived exertion; VAS, visual analogue scale. In A, marks on the line graph indicate the same as in Fig. 1. In B, * indicates the same as in Fig. 1.

Subjective feelings as assessed by rate of perceived exertion (RPE) and visual analogue scale (VAS) are shown in Fig. 5. RPE linearly increased in accordance with increasing gait speed, and a two-way ANOVA revealed a significant main effect of speed and temperature, and interaction. A Bonferrnoi post-hoc test further revealed that RPE at gait speeds above 1.33 m s^−1^ at 33°C was significantly higher compared to the 23°C and 13°C trials (all *P*<0.05). Respiratory fatigue assessed by VAS was also significantly higher at 33°C compared to the other two trials (*P*<0.05) with no differences between the latter two trials. On the other hand, no differences were observed in leg muscle fatigue according to VAS among the conditions (all *P*>0.05).

## DISCUSSION

### Hot versus cool and thermoneutral conditions

We calculated EE by using 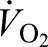 and 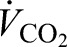 based on previous studies (Horiuchi et al., 2016, 2017). To date, there is a debate whether the 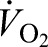 was unchanged in a hot environment (Hayashi et al., 2006; No and Kwak, 2016; Nybo and Nielsen, 2001a; Périard and Racinais, 2015, 2016), and findings are equivocal. These discrepancies may be due to the different study settings, e.g. T_a_, exercise mode, intensity and duration, meaning that it may be difficult to compare our results directly with these previous studies. In contrast, 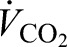 showed higher values in hot conditions, perhaps due to higher values in 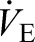 (Nybo and Nielsen, 2001a). Although T_a_ did not affect the overall EE and *Cw* in the present study, a significant interaction was observed in both parameters. This may be caused by a different increasing rate in the EE and the *Cw* at faster gait speeds (see Figs 1D and 4A). Specifically, *Cw* up to the gait speed of 1.17 m s^−1^ was equivalent in all conditions, while above 1.33 m s^−1^, the *Cw* at 33°C was slightly higher. Indeed, the average *Cw* above 1.33 m s^−1^ at 33°C was ∼6% higher compared to the 23°C and 13°C trials (Fig. 4B). In the quadratic function curve (as shown in Fig. 4A; y=*a*x^2^+*b*x+*c*), coefficient *a* controls the degree of curvature of the graph [e.g. a larger magnitude of *a* gives the graph a more closed (sharply curved) appearance]. Therefore, these higher *Cw* values during faster walking at 33°C may affect quadratic function curve in the present study. Mathematically, as the ES in the present study was determined by coefficients *a* and *b* [see Eqn (5) in the Materials and Methods section], a greater coefficient *a* or greater coefficient *b* resulted in a slower ES. We found no significant differences in either coefficient *a* or *b* among the conditions. Coefficient *a* at 33°C was about 26% and 18% greater than the values in the 23°C and 13°C trials, respectively. Meanwhile, coefficient *b* at 33°C was about 18% and 11% less than at 23°C and 13°C, respectively. These results indicate that the increase in coefficient *a* may be a greater factor in determining ES than the increase in coefficient *b*. Indeed, ES at 33°C was ∼7% slower (1.265 at 33°C versus 1.349 at 23°C, and 1.356 m s^−1^ at 13°C), which would be consistent mathematically. These theoretical interpretations may be supported with our previous study (Horiuchi et al., 2016).

Another concern is determining what factors produced the higher *Cw* at faster gait speeds in the 33°C trial. We found a higher HR throughout the walking. It is well known that 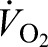 is a functional product of cardiac output (stroke volume×HR) and the *arteriovenous* (*a-v*) O_2_ difference (Karpman, 1975). Although we did not measure stroke volume, a previous study demonstrated that dehydration of ∼1.5% of body mass did not cause significant reductions in stroke volume during submaximal exercise in the heat, compared to a euhydration condition (González-Alonso et al., 2000). The hot environment (T_a_=33°C) in our study reduced body mass only by ∼0.8%, suggesting that stroke volume may not be strongly affected by our experimental protocol. Therefore the slightly, but non-significantly, higher 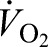 values in the 33°C trial, which resulted in a significant interaction in the *Cw*, can be partly explained by higher HR. Additionally, higher 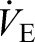 has also been closely linked to excess 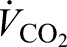 (Schneider and Berwick, 1998). Moreover, it was reported that increases in the *Cw* were attributed to an increased ventilatory cost during walking in patients with chronic heart failure (Figueiredo et al., 2013). Taken together, the slightly but non-significantly higher 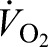 and 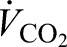 values in the 33°C trial, which resulted in a significant interaction effect in the *Cw*, can be partly explained by higher HR and 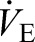.

Nonetheless, the mechanisms underlying the higher 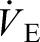 and HR at 33°C remain unclear. It has been frequently observed that 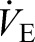 and HR are higher during submaximal exercise in the heat than in a thermoneutral and/or cool environment (González-Alonso et al., 2000; Hayashi et al., 2006; Nybo and Nielsen, 2001a). With regard to 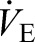 response, one explanation might be related to augmented central command signaling (Nybo and Nielsen, 2001b). Indeed, we found a significantly higher RPE and respiratory fatigue as assessed by VAS at 33°C compared to the 23°C and 13°C trials, which is somewhat consistent with previous findings (Hayashi et al., 2006; Nybo and Nielsen, 2001a; Périard and Racinais, 2016). In terms of HR responses, the elevated HR in the heat has been suggested to be due to enhanced sympathetic nerve activity (SNA) and vagal withdrawal (Jose et al., 1970). Although we did not assess SNA directly, a previous study found that both muscle SNA and HR progressively increased during graded lower body negative pressure in a heat-stressed condition (Cui et al., 2004). Thus, it may be reasonable that higher SNA occurred in the 33°C trial in the present study, resulting in a higher HR during walking. Additionally, it was reported that higher HR is strongly related to higher RPE during exercise in a hot environment (Nybo and Nielsen, 2001b). Therefore, there may still be a possibility that higher RPE as well as higher VAS in respiration may cause higher HR in 33°C compared with 23°C and 13°C. However, we must acknowledge that these potential underlying mechanisms are speculative, and therefore, future studies are required.

Another concern that should be considered may be the effects of relative humidity (RH) on cardiorespiratory responses (i.e. 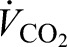, 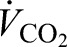, and 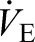). Previous studies reported that the cardiorespiratory system was adequate to fulfil the needs imposed on the participants by an adverse environment even in a high RH condition (Cortili et al., 1996), and that exercise capacity in a warm environment (∼30°C) was impaired at RH >60%, but not impaired at RH <40% (Maughan et al., 2012). These results suggest that the RH of 40% in the present study may not affect cardiorespiratory responses.

### Cool versus thermoneutral conditions

In the present study, we found no differences in any of the physiological or psychological parameters other than cutaneous circulation metrics between the 23°C and 13°C trials. With regard to the effects of cold (cool) exposure on physiological responses including exercise performance, the results of previous studies are controversial. For example, 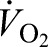 in a cool environment (18°C) was higher than in a thermoneutral (25°C) environment (McArdle et al., 1976), whereas even a severely cold environment of ∼−20°C did not affect peak aerobic capacity (Patton and Vogel, 1984). One previous study investigated cardiorespiratory variables during leg cycling until exhaustion under various T_a_ (∼4–31°C) levels. They found that the exercise time to exhaustion was significantly longer under 11°C compared to other conditions, while 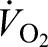 was higher in the order of cold to hot, and there were no differences in HR among environments (Galloway and Maughan, 1997). Although we cannot account for these discrepancies in comparison with our results due to different study settings, it can be said that the cool environmental condition with T_a_ at ∼13°C in the present study may cause insufficient stress to affect cardiorespiratory responses during submaximal walking. It may be worth mentioning that even these cool conditions produced greater individual variance versus thermoneutral conditions, i.e. eight of 14 subjects showed a slowed ES at 13°C, while the ES in the remaining six subjects was faster at 13°C despite the narrow ranges in ES. Future studies are required to investigate these responses under more severe conditions.

### Methodological considerations

There are several concerns regarding the interpretation of our results in the present study. First, we recruited only healthy young males. Thus, it is uncertain whether our results can be generalized to other populations, such as athletes (e.g. speed walkers), females, and the elderly. Further studies are required in the near future. Second, we could not measure the participants' core temperature due to ethical issues, device limitations, and difficulty to recruit participants. While this is a technical limitation, a recent study demonstrated that core temperature showed no differences between 10°C and 30°C during 30 min submaximal leg cycling despite the finding that the T_sk_ in 30°C was higher by ∼8°C than 10°C (Kenefick et al., 2014). In the present study, the magnitude of T_sk_ differences between 13°C and 33°C were also by ∼7°C (Fig. 3). Therefore, it is likely that the core temperature was not affected by our experimental protocols. Finally, we used incremental and continuous walking protocols in the present study. Thus, we could not completely rule out the effect of some form of fatigue (e.g. cardiorespiratory responses and muscle fatigue) on energy cost during walking. However, Poole et al. (1988) have suggested that 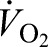 exhibits a delayed quasi-steady state, even more than lactate threshold, and their study employed a higher exercise intensity than the present study. Moreover, the protocol type employed in the present study has been used in some previous studies. (Abe et al., 2017; Horiuchi et al., 2016). Thus, it is likely that our main conclusion may not be significantly affected.

### Future perspectives

The results of the present study may provide useful information on how walking speed should be set to prevent heat-related illness (e.g. hyperthermia, syncope) in a hot environment. Since the ES in the present study was significantly slower in 33°C compared to in 23°C and 13°C, our results may provide applicable recommendations for walking in a hot environment. Recently, Nose and colleagues have reported that brisk walking (e.g. interval walking; repeated three min fast and three min slow walking) can improve physical fitness and reduce a risk of cardiovascular disease, especially in middle-aged people and the elderly (Masuki et al., 2017). However, they also found that adherence to interval walking in young females decreased in summer compared to in winter, at least partially due to a high T_a_ (Tanabe et al., 2018). This may indicate that careful attention should be given when people walk in a hot environment.

## CONCLUSION

Collectively, hot conditions, ∼T_a_=∼33°C, did not affect the overall *Cw* values during walking. However, they slowed the individual ES by ∼7%, while no effect of cool conditions was found on the *Cw* or the ES. From observing HR kinetics, the significantly slower ES at 33°C may be related to a higher HR in the healthy young males used in the present study. Thus, walking in a hot environment with a same intensity (i.e. gait speed) as in a cold or thermoneutral environment should not be recommended, otherwise, walking speed should be slowed at least ∼7% in a hot environment. Taken together, our results can recommend an optimal walking speed for walking in mildly warm environments.

## MATERIALS AND METHODS

### Participants

This study was approved by an ethical committee at the Mount Fuji Research Institute according to the Declaration of Helsinki (No: ECMFRI-03-2014). Before conducting this study, we explained all procedures, possible risks and benefits of participation to the subjects, and all subjects signed an informed consent form. The subjects were 14 male adults with a mean age of 24±5 years, a height of 173±6 cm, and a body mass of 69±10 kg (these values are means±standard deviation [s.d.]). The subjects did not engage in regular exercise. They were free from any known cardiovascular diseases and had not taken any medications.

### Exercise protocols

All experiments were carried out on a motor-driven treadmill (Aero Walker 2200, Combi Wellness Co, Ltd., Tokyo, Japan) and in an environmental chamber (Build-in-chamber, TBR-4, 5SA2GX, Tabai Espec Co, Ltd., Tokyo, Japan). Environmental conditions were set at 23°C, 13°C, and 33°C with a relative humidity of 40%. After emptying their bladders, the subjects were weighed in the nude using a 50 g resolution body weight scale (UC-321, A&D Instruments, Tokyo, Japan). They put on all experimental devices, entered the environmental chamber, and rested in a sitting position for 15 min, followed by 5 min in a standing position on the treadmill for baseline measurements under each environmental condition. Thereafter, each subject began to walk on the treadmill at a level gradient. Based on a slightly modified protocol in our previous studies (Abe et al., 2017; Horiuchi et al., 2016), seven walking speeds were set incrementally at 0.67, 0.83, 1.00, 1.17, 1.33, 1.50, and 1.67 m s^−1^, and each speed lasted for 4 min. After walking, the subjects were weighed again in the nude. Each trial was conducted at the same time of day separated by at least 48 h from the preceding trial, and the trials were completed within one month to avoid changes such as circadian rhythm changes, thermal adaptation, and changes in physical characteristics and fitness level. Moreover, to minimize seasonal effects on sweating during exercise, all studies were completed from September to November. The order of the trials was randomized (i.e. 23°C, or 13°C, or 33°C at seven gait speeds with incremental protocol per day).

### Measurements

Pulmonary ventilation 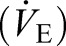 and gas-exchange variables (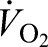 and 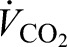) were measured with a computerized breath-by-breath system (AE-310S, Minato Ltd., Osaka, Japan). The standard, known gases (O_2_ 15.23%, CO_2_ 4.999%, and N_2_ balance) and room air were used to calibrate the gas analyzer. Heart rate (HR) was measured throughout the study with a commercial HR monitor (POLAR RC800X, POLAR Electro, Tokyo, Japan). Skin temperature was measured using a commercial thermistor (LT-8, Gram Co. Ltd., Saitama, Japan) at four sites: (1) on the left chest (T_chest_), (2) on the left upper arm (T_arm_), (3) on the left thigh (T_thigh_), and (4) on the left leg (T_leg_). The temperature data were collected every second throughout the study.

Subjective feelings were assessed by two different methods. A modified rate of perceived exertion (RPE) was evaluated at each gait speed in all conditions. The RPE scale was as follows: 0 (nothing at all), 0.5 (very, very weak), 1 (very weak), 2 (weak), 3 (not appreciable; NA), 4 (somewhat strong), 5 (strong), 6 (NA), 7 (very strong), 8 (NA), 9 (NA), 10 (very, very strong). A visual analogue scale (VAS) was also assessed in terms of respiratory and leg muscle fatigue. The VAS consisted of a straight horizontal line of a fixed length of 100 mm. The ends were defined as the extreme limits of the parameter to be measured, oriented from the left (best) to the right (worst). In the present study, we set no perceived dyspnea at all (left) – intolerable dyspnea (right) as the spectrum of respiratory fatigue, and no muscle fatigue at all (left) – intolerable muscle fatigue (right) as the spectrum of muscle fatigue. The subject marked the point on the line that they felt represented their state during walking. The VAS score was determined by measuring in millimeters from the left-hand end of the line to the point that the subject marked.

### Data analysis

As resting baseline values, the average values over the final 2 min of standing were used for all physiological values that underwent continuous measurement, i.e. gas exchange variables, HR, and skin temperature.

To calculate EE at rest and at each gait speed, 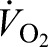 and 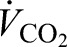 were determined using the following equation.(1)
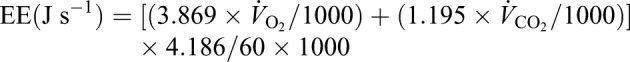
(Brouwer, 1957; Horiuchi et al., 2016, 2017)

At rest, all physiological values (i.e. gas exchange variables, HR, and skin temperature) were averaged over the last 2 min of standing prior to the start of walking. During walking, a single sample with the physiological data averaged over the final 1 min was also obtained.

The following equation was used to calculate each particular *Cw*:(2)
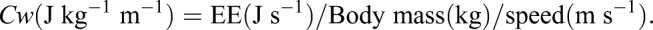
Although their body weight was expected to decrease during walking due to sweating, it was impossible to measure the body weight at all gait speeds. Thus, we used baseline body weight to calculate *Cw* (i.e. pre walking values at each condition).

The *Cw*-v relationship can be mathematically described by the following equation (Horiuchi et al., 2016; Wall-Scheffler and Myers, 2013).(3)
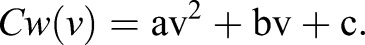
where the constants a, b, and c are determined by the least squares regressions with the actually observed *Cw* values at each gait speed. A differential function of the original quadratic Eqn (2) of each individual can be described as follows:(4)
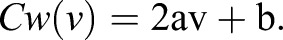
Then, the individual ES was determined at the gait speed when *Cw* (v) was equal to zero; that is, the individual ES could be observed using the following equation:(5)
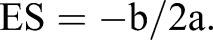
We recently reported that the standing 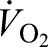 amounted to approximately 50% of the absolute 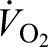 at the level gradient at 0.67 m s^−1^, indicating that care should be taken when calculating *Cw* and ES by subtracting the resting metabolic rate (Abe et al., 2015). Indeed, ES calculated including the resting EE has been found to match the preferred walking speed in many previous studies (Malatesta et al., 2003; Peyrot et al., 2012; Wezenberg et al., 2013; Wall-Scheffler and Myers, 2013). With this background, we included the resting EE when calculating *Cw* and ES.

Mean skin temperature (T_sk_) was calculated using the weighting formula of Ramanathan (1964) as follows:
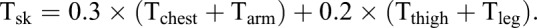


### Statistics

All data are presented as means±s.d. One-way repeated ANOVA was used to compare the baseline values, the individual ES, averaged *Cw* in either slower or faster gait speeds, and VAS. Two-way repeated ANOVA (gait speed×T_a_) was conducted to compare the gas exchange variables, EE, HR, *Cw*, T_sk_, and RPE during walking. A Bonferroni post hoc test for repeated ANOVA was employed when *F* values were significant. All statistical analyses were performed using commercial statistical package software (Sigma Stat ver.3.5, Hulinks, USA). A *P* value less than 0.05 was considered statistically significant.
